# Crocetin Improves Dengue Virus-Induced Liver Injury

**DOI:** 10.3390/v12080825

**Published:** 2020-07-30

**Authors:** Gopinathan Pillai Sreekanth, Aporn Chuncharunee, Pa-thai Yenchitsomanus, Thawornchai Limjindaporn

**Affiliations:** 1 Siriraj Center of Research Excellence for Molecular Medicine, Faculty of Medicine Siriraj Hospital, Mahidol University, Bangkok 10700, Thailand; sreekanthsreebhavan@gmail.com; 2 Department of Anatomy, Faculty of Medicine Siriraj Hospital, Mahidol University, Bangkok 10700, Thailand; achuncharunee@gmail.com

**Keywords:** crocetin, dengue virus-induced liver injury, DENV infection, DENV-induced apoptosis, NF-kB signaling

## Abstract

Dengue virus (DENV) infection is one of the most widespread mosquito-borne viral infections. Liver injury is commonly observed in severe DENV infection, and the present study aimed to examine the efficacy of crocetin treatment in an immunocompetent mouse model of DENV infection exhibiting liver injury. The efficacy of crocetin treatment in DENV-induced liver injury was assessed via both transaminase levels and histopathology analysis. A real-time polymerase chain reaction array was then used to describe the expression of 84 apoptosis-related genes. Using real-time RT-PCR and Western blot analysis, the gene expressions of host factors were investigated. Additionally, the effect of crocetin in NF-kB signaling during DENV infection was studied. We did not observe any significant reduction in virus production when DENV-infected mice were treated with crocetin. However, DENV-infected mice treated with crocetin showed reduced DENV-induced apoptosis. The real-time polymerase chain reaction array revealed pro-inflammatory cytokine expressions to be significantly reduced in the crocetin-treated DENV-infected mice. We also found that crocetin could effectively modulate antioxidant status in DENV-infected mice. Moreover, crocetin demonstrated the ability to reduce the nuclear translocation of NF-kB in DENV-infected mice. Our results suggest that crocetin treatment does not inhibit DENV replication in the liver of DENV-infected mice; however, we did find that crocetin improves host responses that reduce liver injury.

## 1. Introduction

Dengue virus (DENV) belongs to the *Flaviviridae* family, and it comprises four antigenically distinct serotypes (serotypes 1–4). DENV is transmitted to humans by the *Aedes* mosquito [1]. Specific antiviral treatment or a global licensed vaccine against DENV is currently unavailable. The manifestations of DENV infection include symptoms that range from mild dengue fever (DF) to the more severe forms that include dengue hemorrhagic fever (DHF) and dengue shock syndrome (DSS) [2]. The pathogenesis of DENV infection has been extensively investigated; however, it is still not completely understood.

Liver injury is very frequently identified in severe dengue infection [3], and it has been found to be correlated with the clinical pathology [4,5]. An example of this relationship was the observed elevation of aminotransferases. Reactive hepatitis with hepatic failure was also reported in patients with DHF/DSS [6]. The World Health Organization (WHO) has included liver injury as one of the major disease criteria for severe forms of DENV infection [7]. Immunocompetent and immunocompromised mouse models, to study DENV pathogenesis and antiviral strategies, have been reviewed [8,9,10]. In AG129 mice (deficient in the interferon-α/β and -γ receptors), DENV infection resulted in high viral loads in the organs, and led to systemic diseases including liver injury and vascular leakage [11]. In another study, a higher dosage of DENV infection in the AG129 mice resulted in disease pathogenesis, and also resulted in a high mortality rate [12]. However, in immunocompetent mice, the typical signs of liver injury were observed and correlated with elevated liver transaminases [13,14,15,16]. Hepatocyte apoptosis has also been reported in severe dengue cases [17,18], and was significantly observed in the immunocompetent mouse model of DENV infection [19]. Using this animal model, the vital role of mitogen-activated protein kinase (MAPK) signaling in the modulation of DENV-induced apoptosis leading to liver injury was revealed [19]. Our previous studies found that the inhibitors of MAPKs were unable to restrict DENV production, but they improved liver injury by improving host responses, including cellular apoptotic events [20,21,22].

Crocetin is a natural compound that is obtained mainly from the crocus plant (Crocus sativus L.) and *Gardenia jasminoides*. The anti-inflammatory and immunomodulatory properties of crocetin were comprehensively examined in various diseases characterizing liver injury and apoptosis [23]. Crocetin was reported to protect against morphine (opioid analgesic)-induced liver toxicity in the mouse [24]. In an experimental fulminant hepatitis in rats, crocetin treatment was reported to reduce apoptosis, inflammation and oxidative stress responses [25]. Crocetin was also reported to improve post-hemorrhagic shock in patients [26]. In both in vitro and in vivo models of various disease conditions, the pharmacokinetic properties of crocetin were reviewed; however, its effects on DENV infection have not been studied. Accordingly, the present study aimed to investigate the efficacy of crocetin for the treatment of DENV-induced liver injury in a mouse model, and to identify its possible mechanism(s) of action.

## 2. Materials and Methods

### 2.1. DENV Infection and Crocetin Treatment in Mice

The animal experiment protocol was approved by the Siriraj Animal Care and Use Committee (SI-ACUP 019/2555; dated 22 April 2014) under the ethical principles and guidelines of the National Research Council (NRC) of Thailand. Male 8-week-old BALB/c mice were purchased from the National Laboratory Animal Centre (NLAC), Mahidol University, Nakhon Pathom, Thailand. The mice were acclimated in pathogen-free conditions at the laboratory animal facility of the Faculty of Medicine Siriraj Hospital, Mahidol University, Bangkok, Thailand. Intravenous infection with 4 × 10^5^ focus-forming units (FFU) of DENV-2 (strain-16681) via the lateral tail vein was performed. Treatment with crocetin (MP Biomedicals, CA, USA) at a dose of 50 mg/kg was administered via the same route at 1 h before, and 1 h and 24 h after, DENV infection. Other groups of mice that were mock-infected or infected with DENV-2 were treated with 2% dimethyl sulfoxide (DMSO) and used as control groups. Blood samples were collected on the third and seventh days after DENV infection for the hematology analysis and serum preparation. The blood sample collection on day 7 was taken just before the sacrifice. All study mice were euthanized with an intraperitoneal injection of sodium pentobarbital. The liver tissue sample from the experimental mice was harvested, sectioned and immediately stored in RNA later solution (Invitrogen, CA, USA) as per the manufacturer’s protocol.

### 2.2. Hematology and Liver Transaminases

Blood samples were collected on day 7 into an EDTA-containing vacutainer tube (BD Vacutainer^®^ Blood Collection Tubes, NJ, USA). The hematology analysis was performed using a CELL-DYN^TM^ 3700 automated hematology analyzer (Abbott, Chicago, IL, USA). For the preparation of serum, blood samples were allowed to clot and then centrifuged at 2000× *g* for 10 min. The liver transaminases were estimated using a Roche/Hitachi Model 902 chemistry analyzer (Roche Diagnostics, Rotkreuz, Switzerland).

### 2.3. Histopathology Analysis

Liver tissues that were harvested from study mice were fixed in 10 formalin in PBS. The liver tissues were thoroughly washed and paraffin-embedded. The paraffin-embedded blocks were sectioned and mounted on a glass slide for hematoxylin and eosin (H&E) staining. Results were obtained from 6 mice/group and the data was presented as a representative from each group.

### 2.4. DENV-NS1 Viral RNA Quantification from the Liver

DENV-NS1 viral RNA was quantified from the liver tissue using a previously reported protocol [22]. Briefly, the total RNA from the liver tissue homogenates was prepared using a ZR Viral RNA Kit (Zymo Research, USA) and Invitrap Spin Universal RNA Mini Kit (Stratec Molecular, Birkenfeld, Germany), respectively. An in vitro transcription-derived DENV-NS1 RNA with known copies was 10-fold serially diluted (standard) and the samples prepared from the liver homogenates simultaneously underwent qRT-PCR in a Roche LightCycler 480 Instrument (Roche Applied Science, Penzberg, Germany). The Ct values of the serially diluted standard were used to plot a standard curve and were compared with the test samples. The Ct values were analyzed for determining the DENV NS1 copies in the test samples. Data were obtained from 8 mice/group. Results were represented as virus copies/µg of total RNA obtained.

### 2.5. Focus-Forming Unit (FFU) Assay in Liver Homogenates

FFU assay was performed to count the viral titers in the livers of the study mice, as previously described [22]. Briefly, liver tissue samples were homogenized in Roswell Park Memorial Institute (RPMI) Medium and centrifuged at 14,000 revolutions per minute (RPM) for 12 min to obtain a clear supernatant. The obtained supernatant was filter-sterilized and used to determine the viral titer by FFU assay. Data were obtained from 6 mice/group and presented as FFU/mg of the liver tissue sample.

### 2.6. Superoxide Dismutase and Catalase Activity

The enzyme activity of superoxide dismutase (SOD) and catalase (CAT) was evaluated using commercial kits purchased from Abcam (Cambridge, UK) (ab65354 and ab83464, respectively). Liver homogenates were prepared from at least 6 mice/group, in a lysis buffer, and centrifuged at 14,000× *g* for 5 min at 4 °C to obtain a supernatant. The experiments were performed with the obtained supernatant according to the manufacturer’s instructions. The absorbance of the SOD and CAT reaction mixtures was read at 450 nm and 570 nm, respectively, using a Synergy™ microplate reader (Bio-tek Instruments, Inc., Winooski, VT, USA).

### 2.7. Gene Expression Profiler (RT-PCR Array)

RNA samples from the livers of study mice were prepared using an Invitrap Spin Universal RNA Mini Kit (Stratec Molecular, Birkenfeld, Germany). Equal concentrations of RNA samples were reverse-transcribed to cDNA using the SuperScript^®^ III First-Strand Synthesis System (Invitrogen, CA, USA). The obtained cDNA was mixed with SYBR Green RT^2^ qPCR Mastermix (QIAGEN, Hilden, Germany) and aliquoted into an RT^2^ Profiler™ PCR Array (Qiagen, Hilden, Germany) containing 84 selected apoptosis-related genes. PCR amplification was performed in a Roche LightCycler 480 Instrument (Roche Applied Science, Rotkreuz, Switzerland), and Ct values were analyzed using the ‘Qiagen Data Analysis Center’ web platform (2^–Ct^ analysis).

### 2.8. RT-PCR Analysis

RNA was prepared from the livers of study mice and quantified using a Nanodrop spectrophotometer (Thermo Fisher Scientific, Waltham, MA, USA). Equivalent concentrations of RNA samples were prepared and converted to cDNA. The obtained cDNA was mixed with LightCycler^®^ 480 SYBR Green Mastermix (Invitrogen, CA, USA) and the primer set for the individual gene of interest. The primer blast program was used for customized oligo designs that were used in the current study and the designs are shown in Table 1. RT-PCR reactions were performed in a Roche LightCycler 480 Instrument (Roche Applied Science, Rotkreuz, Switzerland), and the obtained Ct values were normalized to the Ct value of glyceraldehyde-3-phosphate dehydrogenase (GAPDH)—the housekeeping gene control. The results were further analyzed by the 2^–Ct^ method and the results of that analysis represent data from at least 6 mice/group.

### 2.9. Cytosolic and Nuclear Fractionation of Proteins from Liver Tissues

Liver tissue samples were homogenized and fractionated to cytosolic and nuclear fractions using a Subcellular Protein Fractionation Kit for Tissues (Thermo Fisher Scientific, Waltham, MA, USA). Samples from 6 mice/group were used in the experiments. Briefly, the stored frozen tissues were thawed and homogenized in protease inhibitor-containing buffers. The cytoplasmic and nuclear fractions were separately collected by centrifugation according to the manufacturer’s instructions. The concentration of the cytoplasmic and nuclear protein fractions obtained were estimated and stored at −70 °C.

### 2.10. Western Blot Analysis

Protein samples that were prepared from the liver tissue homogenates were separated by sodium dodecyl sulfate-polyacrylamide gel electrophoresis (SDS-PAGE), blotted onto a nitrocellulose membrane, and blocked with 5% bovine serum albumin (BSA) or 5% skim milk for an hour to prevent non-specific binding. The nitrocellulose membrane was incubated overnight with mouse-anti-glutathione peroxidase 1/2 (Gpx 1/2), or rabbit anti-total JNK1/2, or mouse anti-phosphorylated JNK1/2, or mouse anti-total p38 or mouse anti-phosphorylated p38, or rabbit anti-cleaved caspase-3 or mouse anti-heamoxygenase-1 (HO-1), or mouse anti-cycloxygenase-2 (COX-2), all of which were purchased from Cell Signaling Technology, Inc. (Danvers, MA, USA). The membrane was further incubated in the dark at room temperature with the corresponding horseradish peroxidase (HRP)-conjugated secondary antibody (Dako Denmark A/S, Glostrup, Denmark). Immune complexes were detected by enhanced chemiluminescence (SuperSignal West Pico Chemiluminescent Substrate; Thermo Fisher Scientific, Waltham, MA, USA). GAPDH was used as the housekeeping gene control to normalize the experiments.

To characterize the nuclear translocation of NF-kB, the proteins obtained from the nuclear and cytoplasmic fractions were separately subjected to Western blot analysis using rabbit anti-NF-kB p65 or mouse anti-NF-kB p50 primary antibodies. The corresponding horseradish peroxidase (HRP)-conjugated secondary antibody (Dako, CA, USA) was used to detect the immune complexes. GAPDH and Lamin B1 were used as the housekeeping gene controls for cytosolic and nuclear fractions, respectively.

The immunoblots obtained from 6 mice/group underwent densitometry analysis using the ImageJ program (United States National Institutes of Health, Bethesda, MD, USA), and the image intensity was reported.

### 2.11. Statistical Analysis

The results obtained were analyzed by one-way analysis of variance (ANOVA) followed by Bonferroni post-hoc test using GraphPad Prism software (GraphPad Software, Inc., San Diego, CA, USA). A *p*-value of less than 0.05 was considered to be statistically significant.

## 3. Results

### 3.1. Crocetin Did not Reduce Dengue Virus Production in the Liver of DENV-Infected Mice

**Figure 1:**
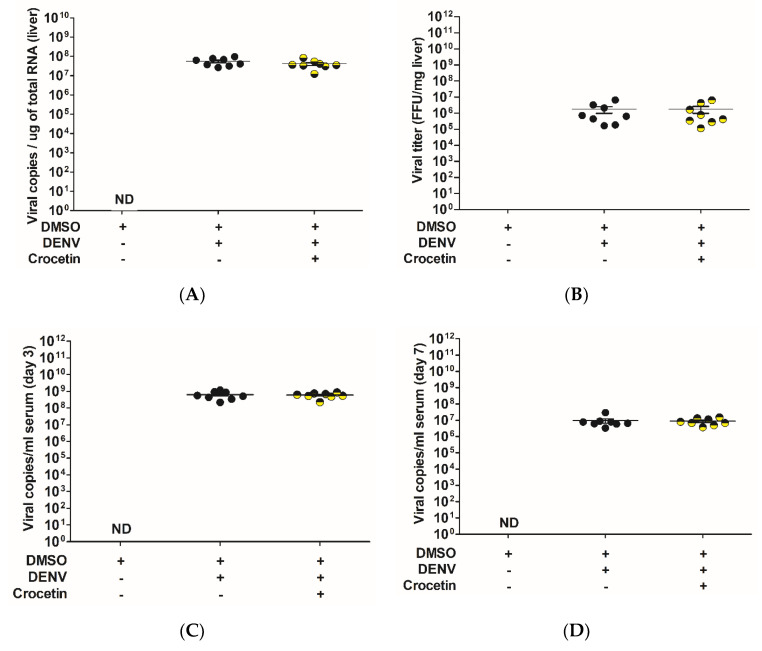
Crocetin did not reduce dengue virus replication in DENV-infected mice. BALB/c mice were infected with DENV-2 (4 × 10^5^ FFU/mL) and then treated with crocetin (50 mg/kg) dissolved in 2% DMSO. DENV-infected 2% DMSO-treated and mock-infected 2% DMSO-treated groups of mice were used as positive and negative controls, respectively. DENV NS1 viral RNA in liver tissue was quantified using an in vitro-derived viral standard (standard curve method). A standard FFU assay was also conducted with liver homogenates. The DENV NS1 viral RNA quantification in the serum on day 3 and day 7 post-DENV infection was also estimated. The results are given, as follows: (**A**) DENV NS1 viral RNA quantification in the liver; (**B**) FFU assay from liver homogenates; (**C**) DENV NS1 viral RNA quantification in the serum on day 3 after DENV infection; (**D**) DENV NS1 viral RNA quantification in the serum on day 7 after DENV infection.

The effect of crocetin treatment in the liver of DENV-infected mice was analyzed using an in vitro transcription derived from viral NS1 copies with known copy numbers. A standard curve was plotted using the Ct values obtained from the 10-fold dilutions of known NS1 copies used in the assay. The Ct values of the liver samples were compared with the standard curve to obtain the exact number of viral NS1 copies. In the liver, we did not find any significant differences in the copy numbers between the DENV-infected control mice and the DENV-infected mice that were treated with crocetin (Figure 1A). Further, to confirm the viral quantification in the liver, an FFU assay was conducted with the liver tissue homogenates. The results obtained from the FFU assay (Figure 1B) also supported the viral NS1 quantification, as no reduction in the viral titer was observed.

Similar observations were seen in the serum samples on day 3 and day 7, as there was no significant reduction in the viral titer between the control and crocetin treated group (Figure 1C,D). The virus titer in the serum samples was found to be much higher on day 3 (Figure 1C); however, the viremia in the serum samples was subsequently reduced on day 7 (Figure 1D).

### 3.2. Crocetin Improved DENV-Associated Clinical Manifestations in Mice

**Figure 2:**
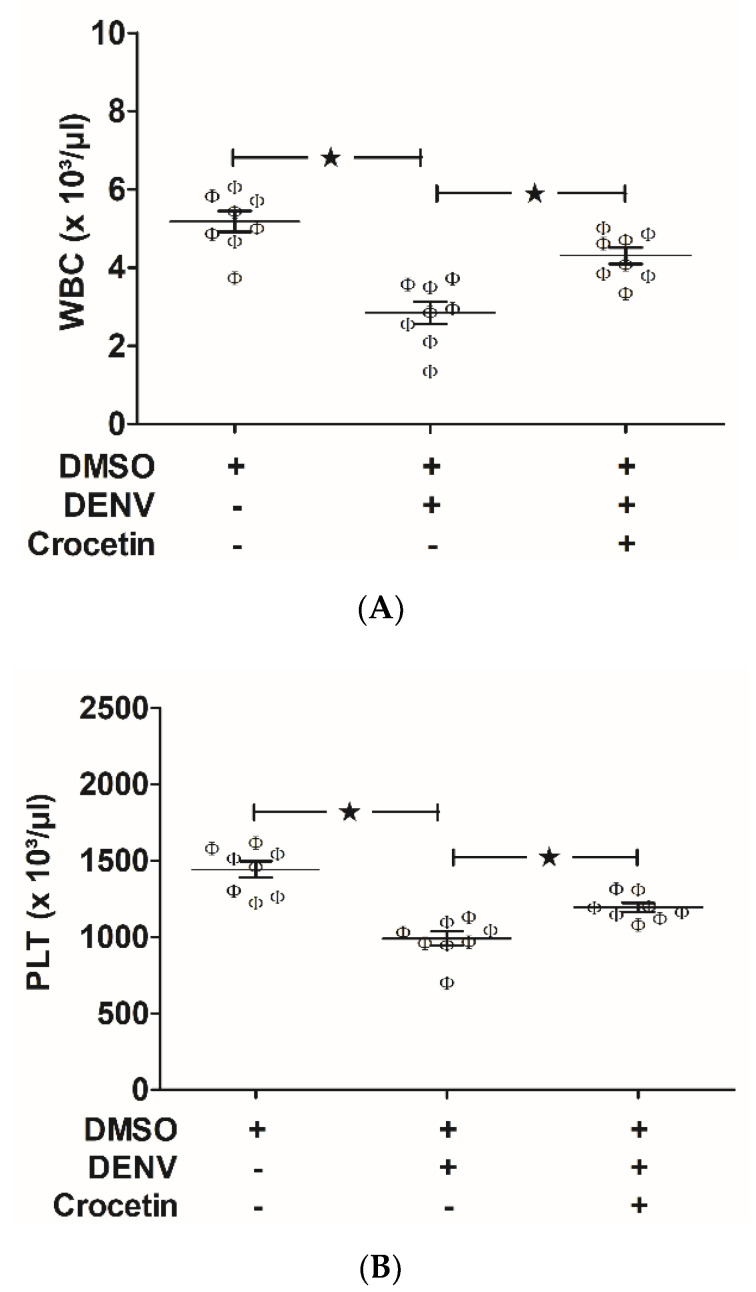
Crocetin improved leucopenia and thrombocytopenia in DENV-infected mice. BALB/c mice were infected with DENV-2 (4 × 10^5^ FFU/mL) and treated with crocetin (50 mg/kg) dissolved in 2% DMSO. DENV-infected 2% DMSO-treated and mock-infected 2% DMSO-treated groups of mice were used as positive and negative controls, respectively. Blood samples were collected on day 7 post-infection for clinical hematology analysis. White blood cell (WBC) count (**A**) and platelet (PLT) count (**B**) are shown. The black star represents, those groups were compared and found to be significant.

White blood cell (WBC) and platelet (PLT) counts were found to be decreased in DENV-infected mice (Figure 2A,B), which suggests that DENV infection in mice causes leucopenia and thrombocytopenia. Crocetin treatment at a dosage of 50 mg/kg improved these DENV-associated clinical indications in mice (Figure 2A,B), which suggests the efficacy of crocetin for modulating DENV-induced leucopenia and thrombocytopenia in mice.

### 3.3. Crocetin Improved Liver Injury in DENV-Infected Mice

**Figure 3:**
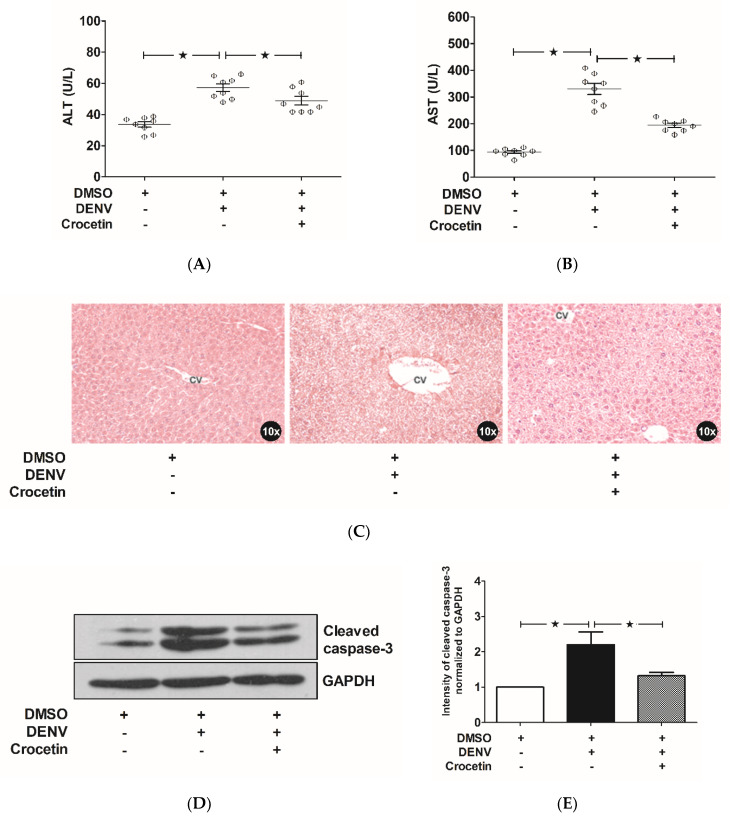
Crocetin reduced liver injury in DENV-infected mice. BALB/c mice were infected with DENV-2 (4 × 10^5^ FFU/mL) and then treated with crocetin (50 mg/kg) dissolved in 2% DMSO. DENV-infected 2% DMSO-treated and mock-infected 2% DMSO-treated groups of mice were used as positive and negative controls, respectively. Blood samples were collected on day 7 post-infection, and serum was prepared for the estimation of liver enzymes. Liver tissues were excised after sacrifice on day 7. Histopathologic analysis with H&E staining was then conducted. Western blot analysis was performed on protein samples obtained from liver homogenates, and the blots obtained were quantitated by ImageJ densitometry analysis. The results are shown as follows: (**A**) AST, (**B**) ALT, (**C**) Histopathologic analysis with H&E staining (CV represents the central vein), (**D**) Western blot analysis of cleaved caspase-3 expression, and (**E**) Densitometry of cleaved caspase-3 expression. The black star represents, those groups were compared and found to be significant.

Liver transaminases (aspartate aminotransferase (AST) and alanine aminotransferase (ALT)) in the serum of the study mice were estimated in order to evaluate for liver injury. The DENV-infected control mice showed significant elevations of both AST and ALT (Figure 3A,B), compared to the levels observed in uninfected mice. Interestingly, the levels of AST and ALT were found to be reduced in the DENV-infected mice that were treated with crocetin (Figure 3A,B). When compared to the uninfected mice, the DENV-infected mice treated with crocetin still presented elevated transaminases; although, these were comparably less than those of the DENV-infected control mice. Liver histopathology analysis revealed typical signs of liver injury in DENV-infected mice not treated with crocetin (Figure 3C). Briefly, dilated sinusoid capillaries were seen with Kupffer cell hyperplasia around the areas. Cytoplasmic vacuolization was seen with enlarged hepatocytes. Cellular necrosis and apoptosis were seen very noticeably. The presence of inflammatory cells and ballooning of the hepatocytes were extensively seen in DENV-infected mice, whereas the level of injury was found to be less pronounced in DENV-infected mice treated with crocetin (Figure 3C). DENV-infected mice treated with crocetin presented with a lesser hepatocyte ballooning compared to that of the DENV-infected control. The sinusoids were rigid, with a lesser degree of cell death when DENV-infected mice were treated with crocetin. The expression of cleaved caspase-3 was found to be higher in the liver samples of DENV-infected mice than in the samples harvested from uninfected mice (Figure 3D); however, when DENV-infected mice were treated with crocetin, the expression of cleaved caspase-3 was found to be significantly reduced (Figure 3E). This finding suggests the efficacy of crocetin for modulating DENV-induced liver injury via decreased hepatic cell apoptosis.

### 3.4. Crocetin Reduced Apoptosis in the Livers of DENV-Infected Mice

To preliminarily investigate the mechanism of action by which crocetin improves hepatic cell apoptosis in the livers of DENV-infected mice, a PCR array profiler was used. The gene expression profiles of 84 apoptosis-associated genes were simultaneously analyzed in a single assay platform. The expression profiler was normalized to β-actin, the housekeeping gene control. The most significant fold changes that too place in uninfected control mice are shown in Table 2.

**Figure 4:**
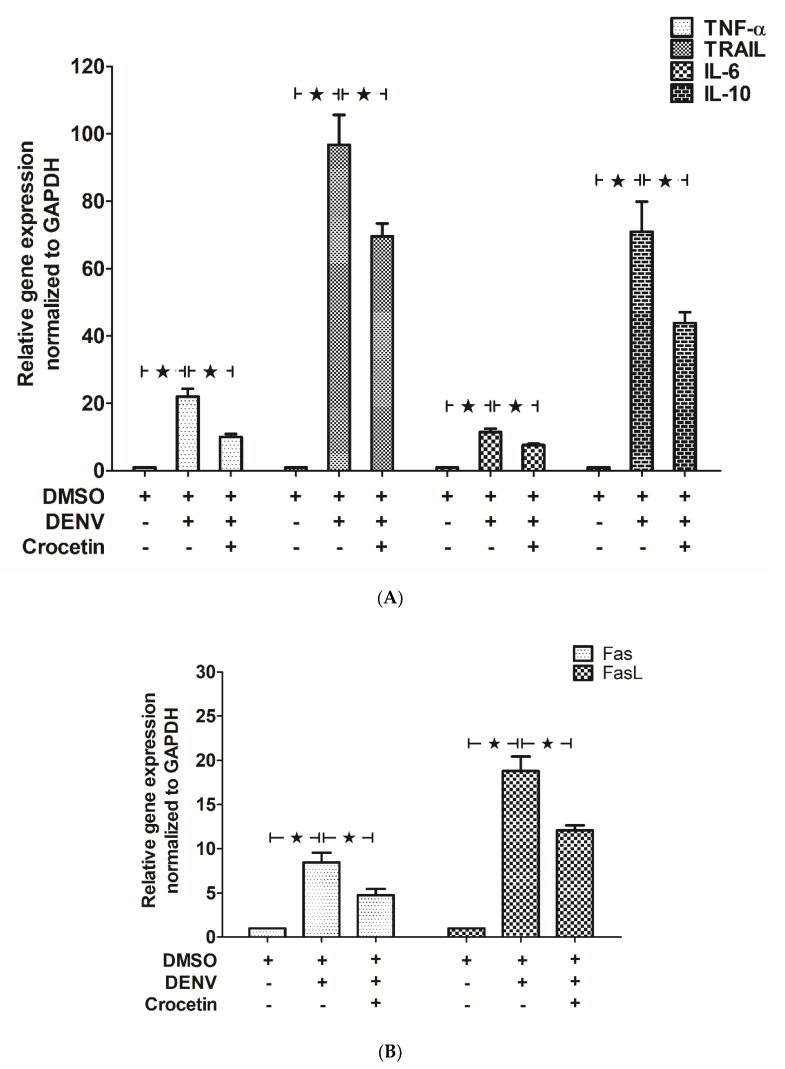
Crocetin reduced the expression of inflammatory cytokines and pro-apoptotic mediators in the livers of DENV-infected mice. BALB/c mice were infected with DENV-2 (4 × 10^5^ FFU/mL) and then treated with crocetin (50 mg/kg) dissolved in 2% DMSO. DENV-infected 2% DMSO-treated and mock-infected 2% DMSO-treated groups of mice were used as positive and negative controls, respectively. RNA samples prepared from liver tissues were converted to cDNA. RT-PCR was conducted, and the Ct values were normalized to that of GAPDH. The mRNA expressions of (**A**) *TNF-α*, *TRAIL, IL-6* and *IL-10,* and (**B**) *Fas* and *FasL*, are shown. The black star represents, those groups were compared and found to be significant.

Selected genes from the profiler, including Tumor Necrosis Factor-α (*TNF-α*), Fas, Fas-Ligand (*Fas-L*) and Interleukin 10 (*IL-10*), were examined using the designed primer set shown in Table 1. In addition to the list of genes from Table 1, the expressions of prominent pro-inflammatory cytokines, including Interleukin 6 (*IL-6*) and TNF-related apoptosis-inducing ligand (*TRAIL*), were investigated. Higher expressions of pro-inflammatory cytokines, including *TNF-α*, *TRAIL* and *IL-6*, were observed in DENV-infected mice compared to those found in uninfected control mice (Figure 4A). The expression of anti-inflammatory cytokine *IL-10* (Figure 4A) was also found to be elevated in DENV-infected mice. Additionally, the expressions of pro-apoptotic factors, including *Fas* and *Fas-L*, were also found to be elevated in the DENV-infected mice (Figure 4B). Interestingly, the expressions of these genes were significantly decreased in DENV-infected mice treated with crocetin, compared to DENV-infected control mice (Figure 4B).

### 3.5. Crocetin Balanced Antioxidant Enzymes in the Livers of DENV-Infected Mice

**Figure 5:**
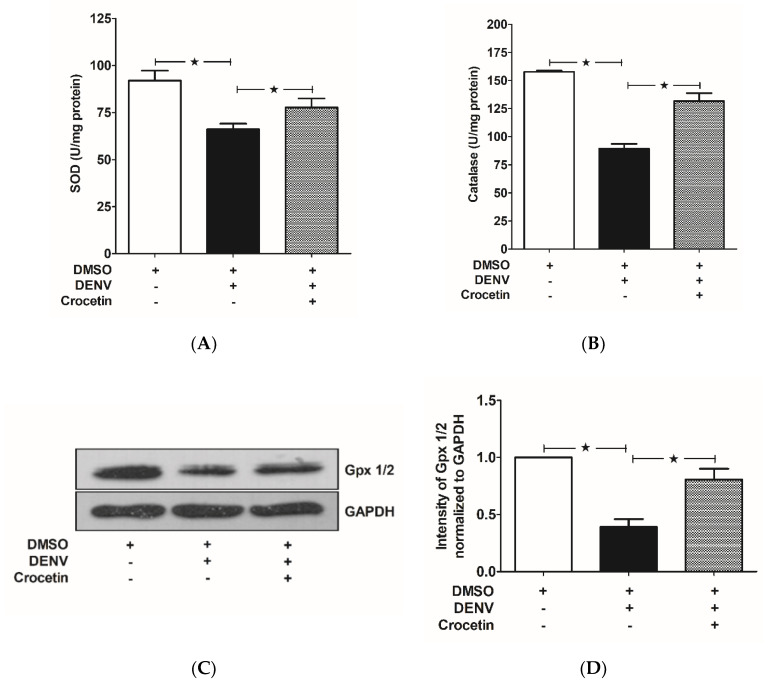
Crocetin reduced oxidative stress in the livers of DENV-infected mice. BALB/c mice were infected with DENV-2 (4 × 10^5^ FFU/mL) and then treated with crocetin (50 mg/kg) dissolved in 2% DMSO. DENV-infected 2% DMSO-treated and mock-infected 2% DMSO-treated groups of mice were used as positive and negative controls, respectively. For the antioxidant enzyme (SOD and catalase) activity assay, protein samples were prepared from liver homogenates using standard protocols. SOD and catalase absorbance were read at 450 nm and 570 nm, respectively. For the expression of Gpx 1/2, Western blot analysis was conducted on protein samples obtained from liver homogenates, and the blots obtained were quantitated by ImageJ densitometry analysis. The results are given as follows: (**A**) SOD activity, (**B**) Catalase activity, (**C**) Gpx 1/2 expression, and (**D**) Densitometry analysis of Gpx 1/2 expression. The black star represents, those groups were compared and found to be significant.

Reduced enzyme activities of both SOD (Figure 5A) and CAT (Figure 5B) were observed in the livers of DENV-infected mice; however, crocetin treatment reversed these enzyme activities (Figure 5A,B) to maintain the redox status in mice. The expression of glutathione peroxidase 1/2 (Gpx 1/2) was found to be reduced in the livers of DENV-infected mice, and this decreased expression was induced by crocetin treatment (Figure 5C,D). These findings demonstrate that immunocompetent mice infected with DENV by intravenous injection exhibited liver injury with reduced activities of antioxidant enzymes, including SOD, CAT and Gpx 1/2. Our findings further suggest the efficacy of crocetin in modulating antioxidant status in DENV-infected mice.

### 3.6. Crocetin Modulated HO-1 and COX-2 Expression in the Livers of DENV-Infected Mice

**Figure 6:**
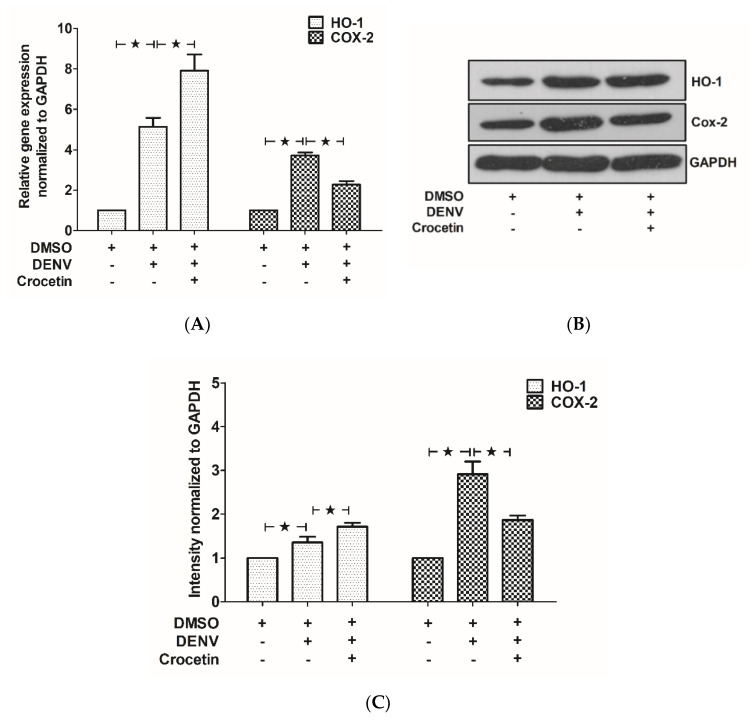
Crocetin increased HO-1 expression, but reduced COX-2 expression in the livers of DENV-infected mice. BALB/c mice were infected with DENV-2 (4 × 10^5^ FFU/mL) and then treated with crocetin (50 mg/kg) dissolved in 2% DMSO. DENV-infected 2% DMSO-treated and mock-infected 2% DMSO-treated groups of mice were used as positive and negative controls, respectively. RNA samples prepared from liver tissues were converted to cDNA. RT-PCR was conducted, and the Ct values were normalized to that of GAPDH. For the protein expressions, Western blot analysis was conducted with protein samples obtained from liver homogenates, and the blots obtained were quantitated by ImageJ densitometry analysis. The result shows (**A**) mRNA expressions and (**B**) protein expressions of HO-1 and COX-2, and (**C**) represents the densitometry analysis of the immunoblots. The black star represents, those groups were compared and found to be significant.

To further understand the host factors in DENV-infected mice treated with crocetin, mRNA (Figure 6A), and protein expressions (Figure 6B,C) of HO-1 and COX-2, were analyzed.

A higher expression of HO-1 was observed in DENV-infected mice compared to that of the uninfected control mice. Interestingly, HO-1 was found to be much more highly expressed in the DENV-infected mice treated with crocetin (Figure 6A–C). COX-2 expression was significantly upregulated in DENV-infected mice; however, this expression was found to be decreased when DENV-infected mice were treated with crocetin (Figure 6A–C).

### 3.7. Crocetin Reduced Phosphorylated JNK and p38 in the Livers of DENV-Infected Mice

**Figure 7:**
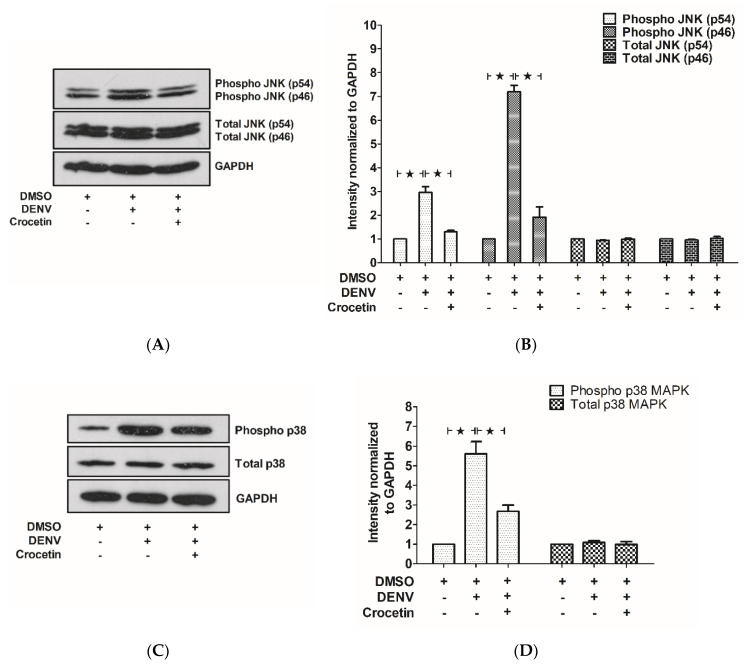
Crocetin reduced the phosphorylation of JNK and p38 in the livers of DENV-infected mice. BALB/c mice were infected with DENV-2 (4 × 10^5^ FFU/mL) and then treated with crocetin (50 mg/kg) dissolved in 2% DMSO. DENV-infected 2% DMSO-treated and mock-infected 2% DMSO-treated groups of mice were used as positive and negative controls, respectively. Protein samples were prepared from liver tissues, and then Western blot analysis was performed with normalization to that of GAPDH. Densitometry analysis of blots was conducted using ImageJ software normalized to individual GAPDH. (**A**) Western blot analysis of phosphorylated and total JNK expressions. (**B**) Densitometry analysis of phosphorylated and total JNK expressions. (**C**) Western blot analysis of phosphorylated and total p38 MAPK expressions. (**D**) Densitometry analysis of phosphorylated and total p38 MAPK expressions. The black star represents, those groups were compared and found to be significant.

Liver tissues were homogenized to prepare the protein samples that were used to determine the effect of crocetin on the phosphorylation of JNK1/2 and p38 MAPK. The phosphorylated forms of both JNK1/2 (Figure 7A,B) and p38 (Figure 7C,D) were significantly elevated in the liver protein samples of DENV-infected mice; however, these phosphorylation events were observed to be reduced upon crocetin treatment (Figure 7A–D). These results suggest the efficacy of crocetin for modulating MAPK signaling in the livers of DENV-infected mice.

### 3.8. Crocetin Reduced Nuclear Localization of NF-kB in the Livers of DENV-Infected Mice

**Figure 8:**
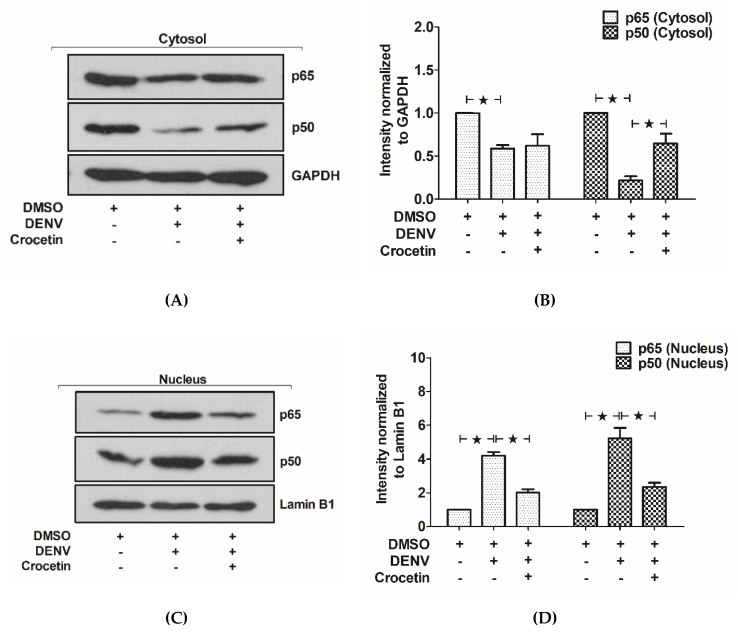
Crocetin reduced the nuclear translocation of NF-kB in the livers of DENV-infected mice. BALB/c mice were infected with DENV-2 (4 × 10^5^ FFU/mL) and then treated with crocetin (50 mg/kg) dissolved in 2% DMSO. DENV-infected 2% DMSO-treated and mock-infected 2% DMSO-treated groups of mice were used as positive and negative controls, respectively. Protein samples were prepared from liver tissues, and cellular fractionation was conducted. The cytosolic and nuclear fractions of the proteins were subjected to Western blot analysis, and the results were normalized to that of GAPDH (cytosolic housekeeping gene) and Lamin B1 (nuclear housekeeping gene). Densitometry analysis of blots was conducted using ImageJ software normalized to the individual housekeeping genes. (**A**) Western blot analysis of NF-kB p65 and p50 from the cytosolic fraction. (**B**) Densitometry analysis of NF-kB p65 and p50 from the cytosolic fraction. (**C**) Western blot analysis of NF-kB p65 and p50 from the nuclear fractions. (**D**) Densitometry analysis of NF-kB p65 and p50 from the nuclear fractions. The black star represents, those groups were compared and found to be significant.

The cytosolic and nuclear fractions of proteins obtained from liver tissue were used to estimate the expression of NF-kB. Reductions of the cytosolic NF-kB p65 and NF-kB p50 were observed in DENV-infected mice (Figure 8A,B). Interestingly, the expressions of NF-kB p65 and NF-kB p50 were found to be reversed when DENV-infected mice were treated with crocetin. A higher expression of both NF-kB p65 and NF-kB p50 in nuclear fractions was observed in DENV-infected mice, suggesting NF-kB nuclear translocation. Interestingly, this translocation was significantly reduced in DENV-infected mice treated with crocetin. Our results suggest the ability of crocetin to reduce nuclear NF-kB translocation in the livers of DENV-infected mice.

**Figure 9:**
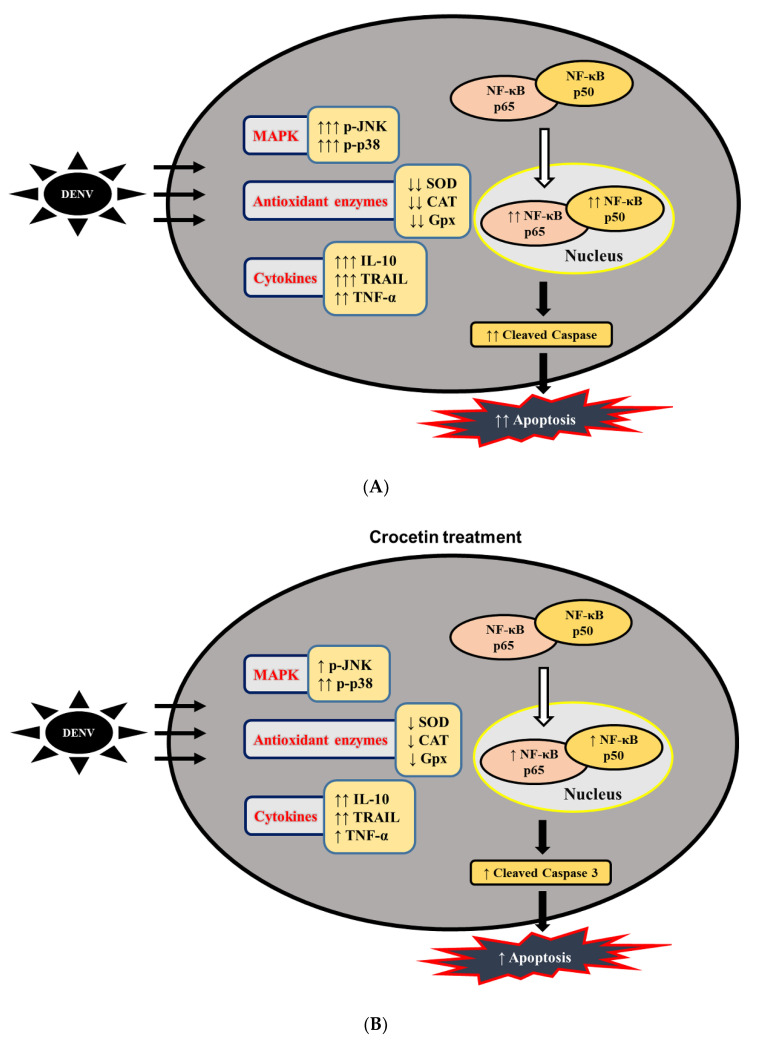
Flow diagram explaining the proposed mechanism of action of crocetin treatment in DENV-infected mice. (**A**) without crocetin treatment, and (**B**) with crocetin treatment.

A graphical representation of the proposed efficacy of crocetin treatment in DENV-infected mice is shown in Figure 9.

## 4. Discussion

Liver injury is most commonly observed in the severe forms of dengue infection [27,28], and hematology parameters were used to predict the severity of disease in dengue patients [29]. DENV-associated clinical indications, including leucopenia and thrombocytopenia, were explained by the previous finding that liver injury was linked with elevated transaminases [30,31]. In immunocompetent mice (Male BALB/c mice of age 8 weeks), DENV-associated clinical symptoms were observed [32,33]. These clinical responses were consistently observed in our experimental model in mice, and crocetin treatment at a dosage of 50 mg/kg of mice improved leucopenia, thrombocytopenia and liver transaminases in DENV-infected mice. Previously, the immunomodulatory and anti-inflammatory properties of crocetin with different dosage regimens were reviewed [34,35]; the dosage of 50 mg/kg was widely used in mice with different disease conditions [36]. The histopathological observations supported the improvements in liver injury when DENV-infected mice were treated with crocetin. In the present study, we injected DENV into mice via the intravenous (IV) tail vein, and treatment with crocetin was also given via the same route, which responded in improving the DENV-induced thrombocytopenia and leucopenia.

We evidenced viral replication in the liver of DENV-infected mice. Similarly, the viral copies in the serum samples on day 3 and day 7 were also seen in DENV-infected mice. In severe DENV-infected patients, a much higher viral load in the liver and serum samples was commonly observed [3,37], which was consistently exhibited in our experimental model of DENV infection in mice. Moreover, our findings lead us to believe the liver is one of the major sites for DENV replication leading to liver injury. Previously, the hepatoprotective effects of crocetin on carbon tetrachloride (CCl_4_)-mediated liver injury, via the modulating of the inflammatory responses, was reported [38]. In an experimentally induced fulminant hepatic failure (FHF) model in rats, crocetin treatment suppressed the pro-inflammatory cytokines, oxidative stress and NF-κB activations [25]. Based on these supportive studies, we aimed to primarily evaluate the effect of crocetin on DENV-induced liver damage. Our research group recently established the effectivity of N-acetyl cysteine (NAC) in reducing viral replication in DENV-infected HepG2 cells, as well as in an immunocompetent mouse model of DENV-induced liver injury [39]. However, the JNK1/2 inhibitor, SP600125, was previously reported to reduce DENV replication in macrophages [40], not in the liver of DENV-infected mice [20]. Our results suggest that crocetin treatment was unable to restrict DENV production in the liver and serum of DENV-infected mice.

In the current study, we applied pre-, co- and post-treatments together, only aiming to identify whether crocetin treatment has any effect on DENV replication or host response, or both. In DENV-infected Huh7 cells, the effects of pre-, co- and post-treatment of sunitinib were explained [41]. Our study in DENV-infected mice has this limitation, and more detailed studies are required to investigate the impact of crocetin treatment with pre-, co- or post-treatments, or their synergistic treatment options, and determine which treatment strategy works the best. That said, we identified that crocetin treatment did not have any direct impact on DENV production in the liver of DENV-infected mice, which suggests the improvements in the liver injury were possibly attained via modulating these host responses.

DENV replication was reported to initiate inflammatory and immunomodulatory responses in the host, causing liver damage [19,42,43,44]. Evidence suggests that thrombocytopenia is associated with inflammatory responses [45], and various oxidative stress-associated proteins were reported to contribute inflammatory responses and liver injury to DENV infection [46,47]. A higher expression of cleaved caspase-3 was observed both in in vitro cultures [48] and in DENV-infected mice exhibiting liver injury [22]. This suggests the vital role of hepatic cell apoptosis in causing DENV-induced liver injury. Our findings are consistent with these findings, and interestingly, we found that DENV-infected mice treated with crocetin displayed a significant reduction in the expression of cleaved caspase-3. Our findings demonstrate the effectiveness of crocetin in restricting hepatic cell apoptosis in DENV-infected mice, and this finding is consistent with our observations of when MAPK inhibitors were used to treat DENV-infected mice [20,21,22]. In an experimental model in rats, the administration of crocetin during resuscitation from hemorrhagic shock improved post-shock survival via the apoptotic pathways [49]. Our results concluded that crocetin treatment did not directly influence DENV production in mice. However, and interestingly, crocetin treatment was able to improve liver injury by restricting hepatic cell apoptosis.

The interplay between apoptosis and oxidative stress in the liver of severe DENV-infected patients was correlated with DENV-associated disease pathogenesis and disease severity [50]. In DENV-infected dendritic cells, regulating the cellular oxidative stress responses was reported to modulate DENV-induced apoptosis [46]. Apoptosis-associated gene profiling, in association with caspase 3 expression, was thoroughly studied in DENV-infected Huh7 cells [51]. In DENV-infected patients exhibiting thrombocytopenia, the effects of oxidative stress responses were previously described [52]. Dendritic cells infected with DENV exhibited higher productions of intracellular reactive oxygen species (ROS) and were reported to cause apoptosis [46]. The major antioxidant enzymes, including SOD, CAT and Gpx, were previously reported to significantly influence the maintenance of redox status in DENV-infected patients [53,54,55]. Imbalance in these antioxidant enzymes contributed to elevated pro-inflammatory cytokines, including TNF-α and interleukin-6 [56]. Our findings are consistent in DENV-infected mice, and crocetin treatment effectively maintained the enzyme activities of these antioxidants. Interestingly, Crocetin was previously reported to improve cardiac stress via the modulation of antioxidant enzymes, including SOD, CAT and glutathione (GSH), and apoptosis was observed to be significantly decreased [57].

In porcine epidemic diarrhea virus-infected cells, ROS production led to apoptosis via the induced phosphorylation of p38 MAPK and JNK [58]. Extrinsic and intrinsic pathways of apoptosis via caspases were previously reported to contribute to p38 MAPK signaling when A549 cells were infected with Newcastle disease virus (NDV) [59]. Our results are consistent with these findings. Specifically, the higher expression of cleaved caspase-3 is the result of a higher phosphorylation of p38 and JNK, which leads to liver injury. In our study, higher expressions of inflammatory cytokines, including TNF-α, TRAIL, IL-6 and IL-10, as well as the pro-apoptotic factors, including Fas and Fas-L, were found to contribute to the phosphorylation of p38 and JNK. Higher expressions of pro-inflammatory cytokines were previously reported to be influential in DENV infection and apoptosis [60]. In DENV-infected monocytes, the expression of pro-inflammatory cytokines, including TNF-α and IL-6, was reported to be elevated and influential in the disease progression [61]. TRAIL was also found to be highly expressed in different cell lines when they were infected with DENV [62]. The serum levels of the anti-inflammatory cytokine, IL-10, were significantly higher in the severe DENV-infected patients, and were established as a prognostic marker for severe DENV infection [63]. This observation was similar in different studies [64,65], and similarly, we found a significantly higher expression of IL-10 in DENV-infected mice. The plasma levels of FasL, TRAIL and TNF-α were identified to be crucial pro-apoptotic factors in DENV-infected patients [66]. The higher expression of FasL was also reported to be a marker for the early course of DENV infection [67]. In human primary monocytes, DENV-induced apoptosis was initiated via the death receptor, Fas [68], and interestingly, the interaction of Fas with its ligand (FasL) was reported to initiate TNF-induced apoptosis in vascular endothelial cells [48]. These inflammatory cytokines and pro-apoptotic factors were reported to exhibit a higher phosphorylation of p38 and JNK [20], and our results were in agreement with this study. Crocetin treatment in DENV-infected mice effectuated a reduction in the expressions of pro-inflammatory cytokines, apoptosis, and phosphorylation of p38 and JNK—all of which suggest improvement in liver injury.

NF-kB is one of the major modulators of both inflammatory and immune responses via the canonical or non-canonical signaling pathway. The canonical pathway gets activated through a p65/p50 heterodimer when pathogens or inflammation interrupts the cellular homeostasis [69]. This may lead to the translocation of p65 and p50 into the nucleus [70]. In liver injury, inflammatory cytokines, including TNF-α and IL-6, were reported to initiate apoptotic cell death via caspase activation and JNK phosphorylation, finally resulting in NF-κB translocation [71]. In another study, JNK and p38 signaling were reported to be crucial for the translocation of cytoplasmic NF-kB towards the nucleus [72]. Crocetin was previously identified to moderate the NF-κ p65 signals leading to reduced inflammatory responses in mice [73]. In the current study, we observed the translocation of both NF-κB p65 and NF-κB p50 in the liver of DENV-infected mice. Interestingly, crocetin reduced this nuclear translocation, leading to improvement in liver injury. The vital role of NF-kB in DENV infection was previously explained by the nuclear translocation of NF-kB-induced cytokine production [74], and NF-kB and JNK signaling were reported to be crucial for DENV-induced COX-2 expression, which was previously shown to facilitate DENV replication [75]. Besides, the inhibition of this NF-kB translocation was reported to activate HO-1 [76], which is required for maintaining antiviral immunity [77]. In the present study, we found HO-1 to be induced when DENV-infected mice were treated with crocetin. Therefore, NF-kB may be one of the important proteins that is modulated by crocetin treatment.

## 5. Conclusions

Crocetin treatment did not reduce DENV production in the liver of DENV-infected mice; however, it improved liver injury. Crocetin treatment in DENV-infected mice also reduced the expression of cleaved caspase-3 in the liver, which suggests its ability to moderate DENV-induced apoptosis. These findings suggest the ability of crocetin to regulate host responses in DENV-infected mice, which results in reduced DENV-induced liver injury. Crocetin treatment in DENV-infected mice was able to reduce DENV-induced oxidative stress, pro-inflammatory responses and NF-kB translocation in the liver. Our findings were preliminary and the cross-stack between these mechanisms needs further investigation.

## Figures and Tables

**Table 1:** The primer designs used for the RT-PCR analysis.

Gene	Forward Primer (5′–3′)	Reverse Primer (3′–5′)
*TNF* *-* *α*	CCCCCAGTCTGTATCCTTCT	TTTGAGTCCTTGATGGTGGT
*Fas*	TGTGAACATGGAACCCTTGA	TTCAGGGTCATCCTGTCTCC
*Fas* *-* *L*	CATCACAACCACTCCCACTG	GTTCTGCCAGTTCCTTCTGC
*IL* *-* *10*	CCAAGCCTTATCGGAAATGA	TTTTCACAGGGGAGAAATCG
*IL* *-* *6*	AGTTGCCTTCTTGGGACTGA	TCCACGATTTCCCAGAGAAC
*TRAIL*	GATGTTGGTGCCTGGAGTTT	AAGCAAAGGGCAGAAAGTCA
*HO* *-* *1*	CACGCATATACCCGCTACCT	CCAGAGTGTTCATTCGAGCA
*COX* *-* *2*	GCGAGCTAAGAGCTTCAGGA	TCATACATTCCCCACGGTTT
*GAPDH*	TGAATACGGCTACAGCAACA	AGGCCCCTCCTGTTATTATG
*DENV NS1*	CCGGCCAGATCTGGAGACATCAAAGGAATC	GCCATCAATGAGAAAGGTCTGG

**Table 2:** Expression profiles of selected genes from the apoptosis PCR array analysis.

Gene	Gene Description	mRNA Expression		
*Il10*	Interleukin 10	1	21.6818	11.7071
*Fadd*	Fas (TNFRSF6)-associated via death domain	1	9.9330	2.9395
*Fasl*	Fas ligand (TNF superfamily, member 6)	1	9.5798	3.9933
*Tnfsf10*	Tumor necrosis factor (ligand) superfamily, member 10	1	8.2658	3.8906
*Casp8*	Caspase-8	1	7.7320	2.1019
*Apaf1*	Apoptotic peptidase activating factor 1	1	6.7321	2.7132
*Cd40*	CD40 antigen	1	4.9866	2.8089
*Fas*	Fas (TNF receptor superfamily member 6)	1	4.7654	1.3851
*Dapk1*	Death-associated protein kinase 1	1	4.5824	1.6507
*Pycard*	PYD and CARD domain containing	1	4.4948	1.4728
*Casp3*	Caspase-3	1	4.1892	2.0943
*Aifm1*	Apoptosis-inducing factor, mitochondrion-associated 1	1	4.0007	2.5176
*Tnf*	Tumor necrosis factor	1	3.9013	1.6363
*Traf1*	Tnf receptor-associated factor 1	1	3.5476	2.0208
*Casp9*	Caspase-9	1	3.3287	1.2058
*Casp7*	Caspase-7	1	2.7851	1.2013
*Cd40lg*	CD40 ligand	1	2.8420	1.4075
*Nfkb1*	Nuclear factor of kappa light polypeptide gene enhancer in B-cells 1, p105	1	2.5692	1.3358
*Traf2*	Tnf receptor-associated factor 2	1	1.8351	1.3586
*Api5*	Apoptosis inhibitor 5	1	0.4351	0.7965
*Dad1*	Defender against cell death 1	1	0.3359	0.8467
*Naip2*	NLR family, apoptosis inhibitory protein 2	1	0.1019	0.7728
**DMSO** **DENV** **Crocetin**		+	+	+
		−	+	+
		−	−	+
